# A multi-centre randomized, open-label phase II trial of continuous erlotinib plus gemcitabine or gemcitabine as first-line therapy in ECOG PS2 patients with advanced non-small cell lung cancer

**DOI:** 10.3892/or.2012.1871

**Published:** 2012-06-15

**Authors:** M. MICHAEL, N. PAVLAKIS, P. CLINGAN, R. DE BOER, M. JOHNSTON, S. CLARKE

**Affiliations:** 1 Division of Hematology and Medical Oncology, Peter MacCallum Cancer Centre, East Melbourne; 2 Royal North Shore Hospital, St Leonards; 3 Southern Medical Day Care, Wollongong; 4 Royal Melbourne Hospital, Parkville; 5 Roche Products, Dee Why; 6 Concord Repatriation General Hospital, Concord, Australia

**Keywords:** advanced NSCLC, EGFR, erlotinib, gemcitabine, lung cancer

## Abstract

Erlotinib and gemcitabine are active in NSCLC and have synergy in other cancers. This study investigated the activity and tolerability of this combination as first-line therapy in ECOG PS 2 patients. Chemotherapy-naïve patients with NSCLC, either stage IIIB (with plural effusion) or stage IV, with measurable disease and ECOG PS 2, and adequate organ function were randomized to receive either erlotinib (150 mg/day p.o.) plus gemcitabine (1000 mg/m^2^, days 1, 8, 15, every 4 weeks) in Arm A or gemcitabine monotherapy (Arm B). The primary end-point was progression-free survival. Seventeen patients of a planned 120 patients were randomized (12 males; 16 Caucasians, 4 large cell, 9 adenocarcinoma; 13 former and 1 never smokers); 16 patients received treatment (8 in each arm). The incidence of treatment-related adverse events (AEs) was 8/8 in Arm A and 6/8 in Arm B; most AEs were grade 1 or 2. The most common treatment-related non-hematological AEs were grade 1 or 2 rash (7/8) and diarrhea (7/8) in Arm A. Two patients in Arm A had partial responses, with durations of 16 and 47 weeks, respectively. Overall disease control rate (N=15) was 86% in Arm A versus 50% for the control arm. Erlotinib plus gemcitabine for the treatment of ECOG 2 NSCLC patients warrants further investigation including intermittent erlotinib regimens.

## Introduction

Overexpression of the epidermal growth factor receptor type 1 (EGFR, HER1) has been shown to play a major role in the pathogenesis of a number of malignancies including non-small cell lung carcinoma (NSCLC) (1,2). Erlotinib inhibits the activity of the intracellular receptor-associated HER1/EGFR tyrosine kinase with nanomolar potency (3). Tyrosine kinase inhibition results in reduced tumor cell proliferation and apoptosis (4).

The efficacy of erlotinib as a single agent has been demonstrated in patients with metastatic NSCLC who have had extensive prior therapy (5,6). Efforts to combine erlotinib with platinum-based doublets such as gemcitabine-cisplatin (TALENT) (7), and carboplatin-paclitaxel (TRIBUTE) (8), in chemo-naïve patients, did not show survival benefit compared with chemotherapy alone. The reasons for the lack of synergy between erlotinib and platinum-based doublets are unclear; the interaction may be at the pharmacodynamic level due to the antagonistic effects of erlotinib on the cell cycle relative to cytotoxic agents.

Much research effort in advanced NSCLC has focused on patients with good performance status (ECOG PS 0-1). However, a large cohort of patients with advanced NSCLC has ECOG PS 2, either due to their cancer or to medical co-morbidities. Treatment of such patients is not well defined due to concerns regarding treatment-related toxicities, rapid deterioration of their clinical state and their poor overall survival relative to patients with ECOG PS 0-1. Treatment options include single agent therapy such as vinorelbine, or modified chemotherapy doublets (9–11). More aggressive modified platinum-based doublet chemotherapy in the first-line setting have also demonstrated benefit (12,13), although at a cost of significantly increased toxicity (9). Thus, the optimal regimen for the treatment of ECOG PS 2 NSCLC patients has not been defined and warrants further research.

Gemcitabine is widely used in the treatment of NSCLC both as a single agent or in combination with other therapies; it has been successfully combined with erlotinib in patients with NSCLC and advanced pancreatic cancer with improved efficacy (14,15).

This multi-centre randomized, open-label, phase II study aimed to assess the activity and tolerability of the combination of continuous erlotinib plus gemcitabine, as first-line treatment, in chemotherapy-naïve patients with advanced NSCLC who are ECOG PS2. However, due to low recruitment and the release of new clinical data on the utility of chemotherapy with an intermittent erlotinib schedule (FASTACT study) (14), the study was terminated early. Therefore, descriptive analyses were performed for the safety data and the best overall response, as per RECIST criteria using the per protocol analysis population.

## Materials and methods

In this a multi-center randomized, phase II trial ECOG PS 2 patients with chemo-naïve advanced NSCLC were randomized to receive continuous erlotinib 150 mg/day plus gemcitabine (Arm A) at 1000 mg/m^2^ over 30 min, on days 1, 8 and 15 of a 4-week cycle, for 6 cycles or until disease progression, unacceptable toxicity or withdrawal or gemcitabine alone (Arm B) at 1000 mg/m^2^ over 30 min, on days 1, 8 and 15 of a 4-week for 6 cycles.

Randomization was stratified by disease stage (IIIB/IV) at the start of study treatment, gender (male/female) and smoking status (current/former/never) using a minimization algorithm with a random element incorporated into the assignment (16). Patients who experienced progressive disease entered a survival follow-up phase (for follow-up and additional NSCLC treatment). Subjects who prematurely withdrew from the study treatment phase without documented disease progression entered a follow-up phase (for follow-up on safety, disease progression and quality of life) unless they withdrew consent.

Second-line therapy post-progression was as per institutional practice, in Arm B erlotinib was offered as optional second-line treatment after disease progression. No maintenance therapy was allowed post-response to first-line therapy.

Eligible patients met the following criteria: i) histologically or cytologically documented, locally advanced or metastatic (stage IIIB with pleural effusion or stage IV) NSCLC, ii) Eastern Cooperative Oncology Croup (ECOG) PS 2 iii) measurable disease according to the response evaluation criteria in solid tumors (RECIST) criteria (17), iv) adequate organ function, v) life expectancy of ≥12 weeks and vi) age 18 or older. Patients were excluded if they had: i) prior systemic anti-tumor therapy for advanced disease, ii) unstable systemic disease, or significant metabolic disease/organ dysfunction, or other condition that contraindicated the use of study medications or that might affect the interpretation of the results or render the patient at high risk from complications. All participating patients provided written informed consent. The study was conducted in accordance with local guidelines and in line with the principles of the Declaration of Helsinki and Good Clinical Practice Guidelines. Ethics approval was obtained from the participating institutions.

Tumor response, at the end of every second cycle (every 2 months) was assessed by the investigator as per RECIST criteria. The analysis was based on the best (confirmed) overall response, defined as the best response recorded from the start of trial treatment until disease progression/recurrence (or death). Clinical and laboratory assessments were conducted at baseline and then at regular intervals throughout the study. Adverse events were graded according to the National Cancer Institute Common Terminology Criteria for Adverse Events (NCI-CTCAE) (version 3.0).

The primary end-point was progression-free survival (PFS) and the secondary end-points were to compare the overall response rate (CR + PR), disease control rate (CR + PR + SD), duration of response and overall survival (OS). The analysis was planned to take place 12 months after the last patient was randomized with a planned sample size of 120 patients. However, recruitment over an 11-months period was slow due to lower than expected number of suitable ECOG PS 2 patients. Consequently the study was closed early (February 2009). Only 17 patients were recruited, and of these 16 patients received study treatment; therefore, many of the pre-specified analyses, as detailed in the study protocol, were not performed. Descriptive analyses were performed for the best overall response, as assessed by the RECIST criteria using the per protocol analysis population and for safety data using the safety analysis population (all patients who received at least one dose/infusion and had at least one safety assessment performed at baseline).

## Results

A total of 17 patients were recruited over a period of 11 months across 8 Australian centers; demographics and baseline characteristics are presented in Table I. One patient randomized to Arm B (gemcitabine) withdrew consent following randomization and did not receive study treatment. Therefore, 16 patients received treatment (eight in each arm).

In Arm A, 2 patients completed 6 cycles of both erlotinib and gemcitabine and continued to receive further erlotinib at the completion of the study. Four patients completed 3–5 cycles of the combination treatment. Five patients required dose reductions of erlotinib to 100 mg. One patient in Arm B completed 6 cycles of gemcitabine monotherapy and 3 patients completed 3 cycles of treatment.

Reasons for discontinuation of the regimen in Arm A were death (n=1), adverse event (n=2), progressive disease (n=2) and other (n=3). In Arm B, reasons for discontinuation of gemcitabine were death (n=1), adverse event (n=3), progressive disease (n=2) and other (n=2).

Safety was evaluable in 16 patients. Eight patients in Arm A and 6 in Arm B had at least one, treatment-related adverse event (Table II). Most adverse events were grade 1 or 2. There was no reported interstitial lung disease (ILD). At the time of study termination, the total number of deaths was 4 in Arm A and 5 in Arm B. Three patients in Arm A and 1 patient in Arm B died within 28 days of the last treatment dose. No treatment related death was reported and the most common cause of death was progressive disease.

Efficacy was evaluable in 15 patients; two were excluded as per protocol definition. One patient in Arm A was lost to follow-up before any tumor assessments were performed, and 1 patient in Arm B received no treatment. At the time of study termination, PFS ranged between 7 and 54 weeks in Arm A with one patient remaining progression-free at 54 weeks. In arm B, PFS ranged between 7 and 25 weeks. Efficacy results for each patient are presented in Table III. The best overall response was partial response, observed in two Caucasian patients in study Arm A (Table III).

The first respondent was a 73 year-old female, former smoker, diagnosed with large cell carcinoma of the lung, stage IIIB with pleural effusion. The patient completed 6 cycles of erlotinib plus gemcitabine and continued on erlotinib thereafter. At the time of data analysis the patient had not progressed at 54 weeks with duration of response of 47 weeks. The second responder was a 74 year-old male, former smoker, diagnosed with stage IV squamous cell carcinoma of the lung. The patient received 4 cycles of erlotinib plus gemcitabine and erlotinib was continued until disease progression. Progression-free survival was 24 weeks with duration of response of 16 weeks. No responses were seen in Arm B (Table III). Overall disease control rate was 86% in Arm A and 50% in Arm B. By week 16, three patients in each arm were observed with progressive disease or death.

## Discussion

The study reported here was terminated early due to slower than expected recruitment. This highlights the difficulties of managing this group of patients: which include rapid deterioration due to their malignancy or co-morbidities, physician bias for any treatment in this group, or more recently, a preference for modified combination therapy. Recently, the potential efficacy of an intermittent schedule of erlotinib with combination chemotherapy in PS 0-1 patients has been demonstrated (14). The intermittent schedule takes advantage of the potential for the pharmacodynamic separation of these agents on the cell cycle. Given the small sample size of this study, the efficacy analyses were descriptive and hence no formal comparisons or definitive conclusions can be made. Nevertheless, acknowledging this, it is of note that in the combination arm (erlotinib + gemcitabine) there were two patients with partial responses, which were prolonged and an overall disease control rate of 86% versus 50% for the control arm.

Analysis of the safety population has demonstrated acceptable toxicities, with no new safety signals observed for the erlotinib plus gemcitabine regimen. Most of the observed adverse events were consistent with those previously reported for erlotinib (6,18). Rash was mild in presentation with no grade 3 reported and patients were managed according to local institutional guidelines. While no discontinuations due to rash were reported, dose reductions to 100 mg were required in two patients in the erlotinib plus gemcitabine arm.

Recently, the concurrent administration of gemcitabine plus erlotinib was also assessed in a phase II trial (19) that enrolled chemotherapy-naïve, stage IIIB or IV NSCLC patients, 70 years or older and PS ≤2. Patients were randomised to gemcitabine monotherapy (1250 mg/m^2^, days 1 and 8), erlotinib monotherapy (150 mg po daily) or the combination (1000 mg/m^2^ days 1 and 8 and 100 mg po daily, respectively). The primary end-point was PFS at 6 months. In contrast to the study reported here, out of the 144 patients enrolled, ~27% were PS 2 across the three study arms. The concurrent administration of erlotinib plus gemcitabine, in this study did not provide additional benefit relative to monotherapy with either agent (19).

Pharmacodynamic separation, achieved by sequential administration of a cytotoxic agent followed by erlotinib, has been proposed based on preclinical data (20) and clinically in the second-line setting with docetaxel (21) and pemetrexed (22,23). The sequential versus concurrent administration of gemcitabine with cetuximab (an EGFR monoclonal antibody) was also recently evaluated in a randomized phase II study in the elderly and PS 2 patient population (24). The study demonstrated a 1-year survival rate of 27.3% in the PS2 subgroup who received the concurrent schedule (24).

Another phase II study investigated the sequential administration of erlotinib plus chemotherapy versus chemotherapy alone in unselected, chemotherapy-naïve patients with advanced NSCLC with ECOG PS 0-1 (14). Patients were randomized to receive erlotinib (150 mg/day) or placebo on days 15–28 of a 4-week cycle that included gemcitabine (1,250 mg/m^2^ days 1 and 8) and either cisplatin (75 mg/m^2^ day 1) or carboplatin (AUC 5, day 1). Although no significant difference in overall survival were observed in this study, the intermittent administration of erlotinib following gemcitabine/platinum chemotherapy demonstrated significant improvements in PFS and was well tolerated (14).

Given the recently reported clinical trial data further investigation of the pharmacodynamic separation of gemcitabine and erlotinib is thus warranted in NSCLC patients with ECOG PS 2 and in the elderly. This treatment approach is currently undergoing further investigation in a phase III study in Australia.

## Figures and Tables

**Table I:** Baseline characteristics of the intend-to-treat population.

	Erlotinib + gemcitabine (n=8)	Gemcitabine (n=9)
Median age years (min-max)	75 (73–85)	76 (40–85)
Gender		
Male	5	7
Female	3	2
Smoking status		
Current	1	2
Former	7	6
Never	0	1
Ethnicity		
Caucasian	7	9
Other	1^a^	0
Disease stage of NSCLC at baseline		
IIIB (with pleural effusion)	2	2
Stage IV	6	7
Histology		
Adenocarcinoma	4	5
Squamous cell carcinoma	3	1
Large cell carcinoma	1	3
ECOG PS 2^b^	8	9

a Patient is of Indian ethnicity.

b Combination of comorbidity and rapidly expanding disease burden, fatigue level, reduced appetite, general physical state, pain, lethargy, slow disease progression.

**Table II:** Summary of common treatment-related adverse events (AEs).

	Erlotinib + gemcitabine (n=8)		Gemcitabine (n=8)	
		
	All grade AEs	Grades 3–5	All grade AEs	Grades 3–5
Hematological				
Thrombocytopenia	0	0	2	0
Neutropenia	2	2	1	1
Anemia	1	0	0	0
Non-hematological^b^				
Rash	7	0	0	0
Diarrhea	7	3^a^	0	0
Nausea	3	1	3	0
Fatigue	4	3	3	1
Vomiting	2	0	0	0
Dyspnea	2	2	1	0
Hypomagnesimia	2	0	0	0
Lethargy	3	0	0	0

a All grade 3.

b Non-hematological treatment related AE observed in ≥2 patients in either study arms are listed.

**Table III:** Key efficacy data (per protocol population).

Best overall response	Progression-free survival (weeks)	Overall survival (weeks)
Erlotinib + Gemcitabine (n=7)		
PR	54^b^	54^d^
PR	24	32^d^
SD	38	39
SD	12	13
SD	12	12
SD	13	27^c^
PD	7	7
Gemcitabine (n=8)		
SD	25	25
SD	23	50^c^
SD	19	32^c^
SD	10	12
PD	8	8
PD	7	14
PD	7	21
Not evaluable^a^	10	10

a Patient did not have adequate post-baseline tumor assessment

b Patient remained progression-free at the time of analysis.

c Patient remained alive at the time of analysis.
